# Transforming food waste: how immobilized enzymes can valorize waste streams into revenue streams

**DOI:** 10.1038/s41538-018-0028-2

**Published:** 2018-10-29

**Authors:** Stephanie M. Andler, Julie M. Goddard

**Affiliations:** Department of Food Science, Cornell University, Ithaca, NY 14853 USA

**Keywords:** Immobilized enzymes, Biocatalysis

## Abstract

Food processing generates byproduct and waste streams rich in lipids, carbohydrates, and proteins, which contribute to its negative environmental impact. However, these compounds hold significant economic potential if transformed into revenue streams such as biofuels and ingredients. Indeed, the high protein, sugar, and fat content of many food waste streams makes them ideal feedstocks for enzymatic valorization. Compared to synthetic catalysts, enzymes have higher specificity, lower energy requirement, and improved environmental sustainability in performing chemical transformations, yet their poor stability and recovery limits their performance in their native state. This review article surveys the current state-of-the-art in enzyme stabilization & immobilization technologies, summarizes opportunities in enzyme-catalyzed valorization of waste streams with emphasis on streams rich in mono- and disaccharides, polysaccharides, lipids, and proteins, and highlights challenges and opportunities in designing commercially translatable immobilized enzyme systems towards the ultimate goals of sustainable food production and reduced food waste.

## Introduction

**Fig. 1:**
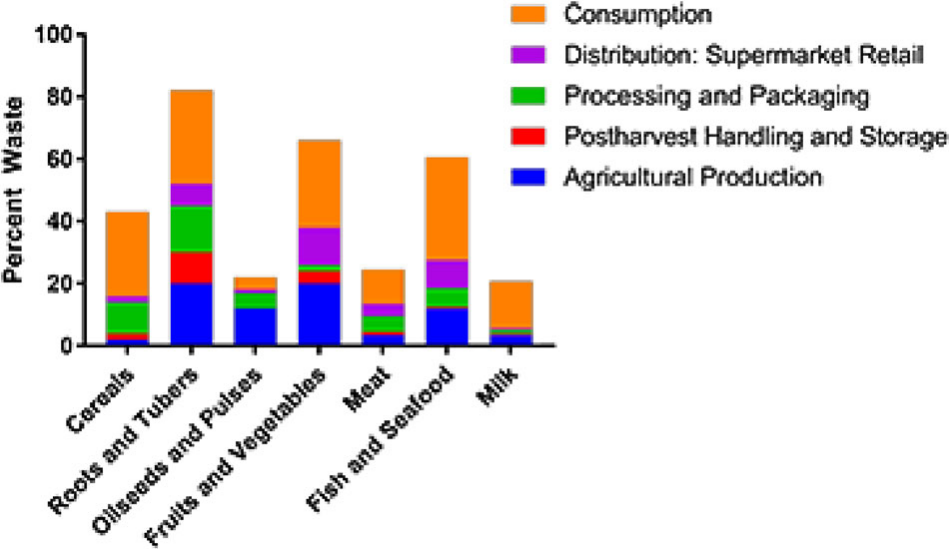
North American food losses and waste by weight percentage for each step of the food supply chain for selected commodities. Percentages are calculated as a percentage of the weight that enters each step^131^

Approximately 40% of all food is wasted,^1,2^ with losses occurring throughout the farm to fork continuum (Fig. 1). While volumes of edible food waste vary geographically, by commodity, and by point in the supply chain, the proportion of food and agricultural waste generated at the pre-consumer level (i.e., post-harvest & processing) represents a significant burden on the environment and remains a global challenge. With the global population expected to reach 9.8 billion by 2050, innovative technological solutions must be developed to reduce food waste, and significant opportunities exist at the food processing level.^3^ Indeed, the high biological oxygen demand of food processing waste streams prevents their direct disposal to wastewater treatment facilities. While the lipids, carbohydrates, and proteins in food and agricultural waste streams are responsible for their high biological oxygen demand, they also have potential for modification into value-added products, thus transforming waste streams into potential revenue streams. Examples of important modifications include oxidation, hydrolysis, acylation, and phosphorylation of carbohydrates; deamination and glycosylation of proteins; and hydrogenation and esterification of lipids. Specifically, esterification reactions are widely employed to manufacture several value-added food and agricultural products. Oils can be esterified with alcohol to produce biodiesel, sugars can be esterified for use as surfactants, starches can be esterified for use as biodegradable plastics, coatings, and hot-melt adhesives,^4,5^ and esterification of flavonoids can enhance their bioavailability and efficacy in promoting health and wellness.^6^ Traditional approaches to these modifications require a chemical catalyst and significant energy input, have limited reaction specificity, and result in formation of by-products, particularly when performed in complex matrices such as food waste streams.

To overcome the limitations of the traditional catalyst systems described above, biocatalysis using enzymes has been explored for chemical modification of agricultural products. For example, waste frying oil can be transformed into biofuels via transesterification, yet because it contains water, this reaction produces soap when a chemical catalyst (e.g., NaOH, Ca(OH)_2_, CaCl_2_) is used. In contrast, the use of lipase for transesterification of waste oil prevents soap formation, reducing byproduct formation. Enzymes also promote the use of greener processing conditions than their chemical counterparts.^7^ Some enzymes (e.g., lipase) have been proven to be active in solvent-free conditions, such as waste cooking oil.^8^ Therefore, enzymes represent a promising alternative to traditional catalysts for upcycling waste streams;^8–10^ their immobilization further improves their recovery, stability, and economic feasibility. Several excellent reviews have been written that outline strategies for waste utilization.^11–14^ Others present specific applications of enzymes to waste streams,^4,15,16^ and other reviews focus on enzyme immobilization technologies.^17–21^ The overall goal of this review was to survey the current state-of-the-art to define opportunities and challenges in valorization of the monosaccharides and disaccharides, polysaccharides, lipids, and proteins present in food and agricultural waste streams using immobilized enzyme systems.

## Enzyme immobilization

While enzymes offer benefits over traditional chemical catalysts in the valorization of waste streams, a major challenge exists in retaining their stability and performance under the conditions typical of food processing waste streams (e.g., elevated temperatures, nonneutral pH). Considering the end application during the design of stabilized enzyme systems is therefore imperative for improving commercial translatability. Indeed, food waste streams are non-ideal environments to enzymes, with conditions outside of their optimum ranges, and enzyme immobilization can enable activity in these non-ideal environments. For example, the pH value of the acid whey produced during the production of strained yogurt is <5, while the optimum pH value of certain glucose isomerases responsible for sweetener production falls between 7 and 8.5.^22^ In addition, while many enzymes have optimal temperature ranges around body temperature (37 °C), their performance at elevated temperatures (50–70 °C) would permit use of greener reaction media (e.g., eutectic mixtures of choline chloride and urea), which are biodegradable and affordable, yet highly viscous at physiological temperatures.^23^ Another challenge to enzyme stability is that some substrates are denaturing to the enzyme itself. For example, lipase can produce biodiesel from alcohols and fatty acids; however, alcohols are known to be denaturing to lipase.^8,24^ Enzyme immobilization onto a solid support or via enzyme–enzyme cross-linking can prevent this structural deformation, thus enhancing performance and stability under the non-ideal conditions typical of food processing waste streams.

Immobilization also enables reuse of the biocatalyst, which can help bridge the gap between the costs of chemical processes and enzymatic processes. However, not only must the enzyme be physically recoverable from the reaction media, but the enzyme must also retain activity over multiple cycles. Choice of carrier material and immobilization method are therefore imperative to rational design of stable, reusable immobilized enzyme systems. Enzyme immobilization techniques such as adsorption, entrapment, encapsulation, and cross-linking have been well reviewed in other work^19,20^ and here we provide a brief summary. While adsorption is a simple and inexpensive method, migration of the enzyme from the surface may occur over time from weak interactions. The variable and often non-ideal pH values of food processing waste streams, as well as the presence of a range of charged components (salts, ions, macromolecules), would increase the likelihood of migration in systems in which the enzyme is adsorbed onto a support via non-covalent interactions, making this technique unsuitable for applications in waste valorization processes. Entrapment and encapsulation offer more protection of the microenvironment of the enzyme. Controlling pore size is critical to prevent enzyme migration while ensuring substrate accessibility, particularly given the complex nature of waste streams that often contain insoluble materials which may hinder substrate access to pores. Cross-linking can provide carrier and carrier-free immobilization, but optimization of the cross-linking parameters is needed to prevent active-site obstruction or denaturation during precipitation. Parameters such as cross-linker selection/concentration, inclusion of inert stabilizing agents, and cross-linking conditions (time, temperature, immobilization in the presence of substrate to protect active site) can be altered to optimize system stability or activity. Indeed, conditions that yield optimal stability are often different from those that yield optimal activity and a compromise must be considered depending on the needs of the intended application. The choice of support material must be carefully considered as well. For example, lipases are known to be hyper-activated by hydrophobic materials.^25^ However, β-galactosidases can lose activity upon immobilization onto hydrophobic materials.^26–28^ Hydrogel beads show promise for activity retention of β-galactosidase, but challenges remain with migration of the enzyme from the matrix.^29^ Another important influence of enzyme immobilization is the potential to shift the optimal working conditions of the enzyme. Indeed, it has been well established that the local pH around enzymes can be manipulated by encapsulation in different polymeric matrices (with cationic materials shifting pH optima more acidic;^30^ anionic materials shifting pH optima more alkaline^31^). Inclusion of stabilizers or stabilizer-mimetic groups (e.g., trehalose, polyethylene glycol) within a carrier or as tethering agents can also influence activity retention.^32^

**Fig. 2:**
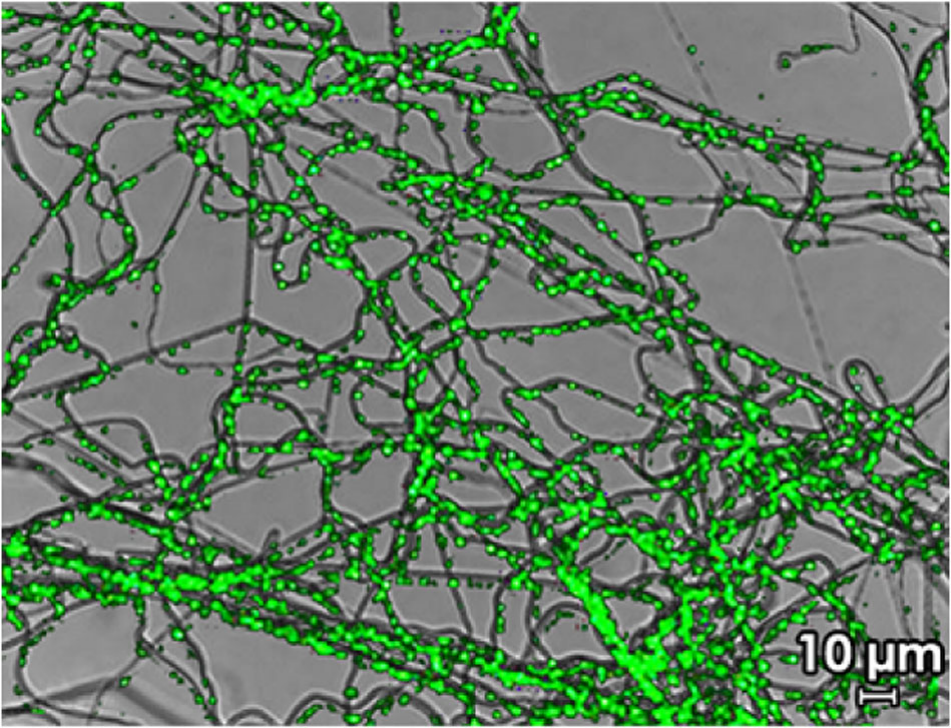
Fluorescein isothiocyanate tagged lactase (green) immobilized in electrospun, hierarchical nanofiber mats. Reprinted from ref.^28^ Copyright (2014), with permission from Elsevier

Nanotechnology offers new approaches to enzyme immobilization, in which enzymes are stabilized on nanostructures such as nanoparticles, nanofibers, and nanorods.^33–35^ As the diameter of the nanoparticle to which the enzyme is tethered decreases, the retained activity of the immobilized enzyme increases due to reduced enzyme–enzyme interactions as well as denaturing interactions between the enzyme and the solid support. Indeed, retained activity of immobilized enzymes that have been bound onto carriers of the order of magnitude of the hydrodynamic radius of the enzyme itself (i.e., nanometers) can approach the activity of native enzyme. However, a practical challenge emerges: in one report, as the size of the particle to which the enzyme was immobilized decreased from 200 to 18 nm, the time required to separate the enzyme-nanoparticle system increased from 60 min to 17 h.^36^ This example highlights the importance of application-driven enzymology research to improve the likelihood of commercial translation of immobilized enzyme systems in food processing waste streams. Hierarchically assembled nanostructures, which present both nano- and macroscale morphologies (Fig. 2), may offer a unique opportunity in improving immobilized enzyme activity retention while permitting improved handling and recovery compared to traditional nanomaterials.^28,37,38^

## Applications of immobilized enzymes in food waste stream valorization

### Carbohydrates

Food processing waste streams rich in carbohydrates are readily amenable to enzymatic valorization by hydrolases and isomerases into value-added products such as sweeteners and prebiotics. Indeed, some of the most profitable, well established processes in food and agricultural systems begin with a carbohydrate substrate; namely, the production of high fructose corn syrup, a $1.7 billion industry in the United States in 2016.^39^ Thermophilic enzymes and their subsequent immobilization, explored in the development of the high fructose corn syrup industry, could be adapted and applied to valorization of food waste streams rich in carbohydrates. Since polysaccharides require hydrolysis before the techniques described for monosaccharides can be used, we discuss carbohydrate waste valorization according to repeat unit characterization, first discussing opportunities for monosaccharides and disaccharides, then opportunities for polysaccharides.

### Monosaccharide and disaccharide carbohydrates

**Fig. 3:**
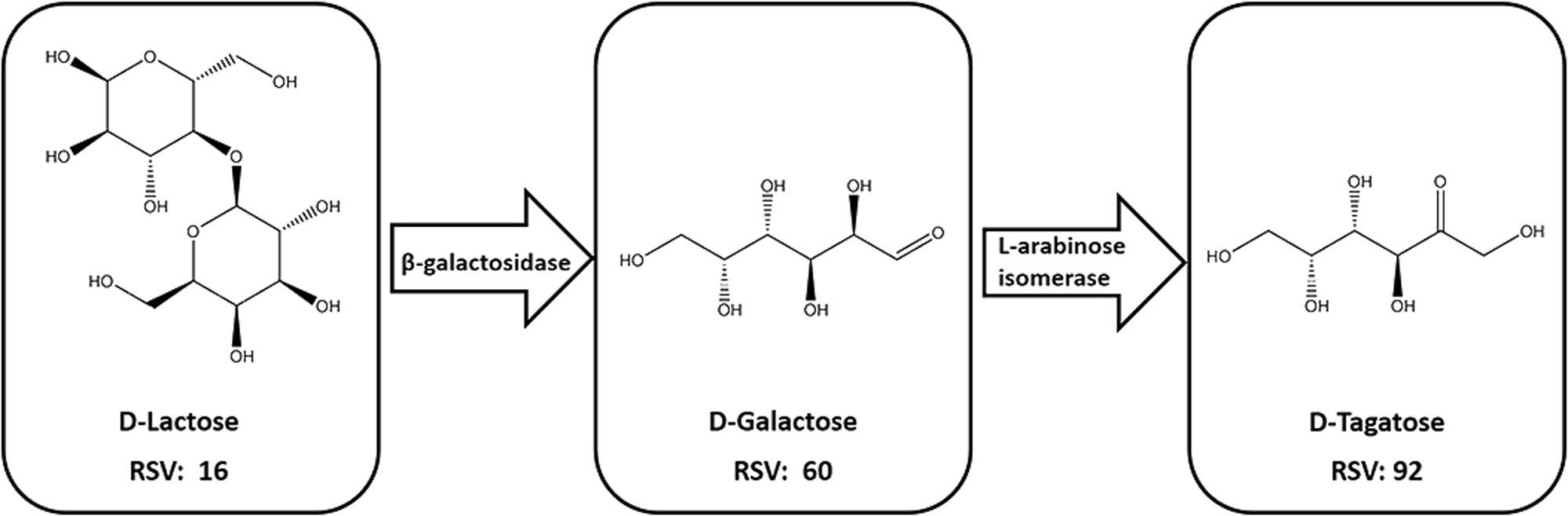
Example of upcycled d-lactose, which has been converted into d-tagatose through a multi-enzyme cascade. d-tagatose has a higher relative sweetness value (RSV) than d-galactose and d-lactose^42,52^

Probably the best example of the value-added reutilization of food processing waste streams is the concentration and fractionation of whey protein from byproduct streams of cheese, casein, and yogurt production. While whey protein is an excellent example of food waste valorization, whey permeate and acid whey (from production of strained yogurt) remain important dairy waste streams rich in the milk sugar lactose, a valuable substrate for enzymatic upcycling.^40^ In 2004, strained, Greek-style yogurt represented only 1–2% of the United States yogurt market. This share of the market increased to nearly 40% in 2015, showing a rapid increase in the production of Greek yogurt, and consequently, the production of acid whey.^41^ Because of the significant volumes of lactose-rich dairy waste streams produced annually, the bulk of the published research on valorization of monosaccharide and disaccharide-rich food waste streams therefore centers on the enzyme-mediated transformation of lactose into value-added products, including sweeteners and prebiotics (Fig. 3). We will therefore focus this section of our review on enzyme-mediated lactose valorization.

With a relative sweetness value of 16% compared to sucrose (100%), lactose is not a valuable sweetener, however upon enzymatic conversion can produce sweeteners with relative sweetness values approaching that of sucrose, some of which have significantly lower caloric value and thus potential additional health benefits. The most common application in lactose valorization is therefore the use of β-galactosidase to hydrolyze waste lactose into glucose (74% relative sweetness) and galactose (30% relative sweetness); this enzyme has also been well studied and is in commercial use for the production of lactose reduce and lactose free milk.^42^ In one of the original reports exploring conversion of waste lactose to sweeteners, Arndt and Wehling^43^ replaced the sucrose in ice cream with hydrolyzed and hydrolyzed-isomerized cheese whey syrup. Immobilized β-galactosidase from *Aspergillus oryzae* transformed the lactose and the authors successfully replaced 25% of the sucrose in the ice cream formulation with few differences in sensory quality. The addition of an immobilized glucose isomerase from *Actinoplanes missouriensis* further transformed the glucose into fructose, and 50% sucrose replacement was achieved. While the secondary isomerization step enabled a sweeter syrup capable of replacing a higher proportion of the sucrose, the low degree of isomerization (34.8%) hindered its economic viability, suggesting the need to improve the yield of the immobilized glucose isomerase system.^43^ Similarly, Lorenzen et al.^44^ studied the addition of enzymatically treated lactose from skim milk, sweet whey, and acid whey to yogurt. The enzymes β-galactosidase (from *Kluyveromyces lactis)* and glucose isomerase (from *Streptomyces rubiginosus*) converted lactose into lactulose, galacto-oligosaccharides, glucose, and galactose, which resulted in a yogurt with enhanced sweetness perception.^44^ More recently, β-galactosidase from *Kluyveromyces lactis* was immobilized in calcium alginate and gelatin spheres cross-linked by combinations of glutaraldehyde and the lectin concanavalin A. When used as the sole cross-linker, concanavalin A enabled more than 70% lactose hydrolysis, and decreased protein leaching from 18 to 3%, suggesting promise of concanavalin A as an enzyme cross-linker.^45^ In other work, a packed-bed reactor was assembled with β-galactosidase from *Kluyveromyces fragilis* immobilized on silanized porous glass. Whey permeate was fed through the reactor and hydrolyzed up to 90% of the lactose, with full activity retention for five 48-h cycles, after which activity began to decline.^46^ These results further support the potential of stabilization of immobilized β-galactosidase for lactose valorization, but highlight the importance of considering hydrolytic stability of tether molecules in preventing enzyme leaching from the support over multiple cycles of reuse.

Recently, rare sugars have garnered increased interest due to their high relative sweetness (70–92%) and low caloric value (0–2 kcal/g) compared to sucrose.^47,48^ Rare sugars are an attractive substitute for sugar alcohols in sugar-free products, because some do not impart a cooling effect or potential gastrointestinal distress as commonly experienced with sugar alcohols.^49^ Some rare sugars, such as tagatose, have other functional benefits, such as slowing the blooming process in chocolate.^50^ Following initial hydrolysis of lactose by β-galactosidase, additional enzymes can be employed to create rare sugars from glucose and galactose. Extensive work by the Izumori group outlined pathways to produce rare sugars.^51,52^ Since numerous pathways exist to produce rare sugars, and research is still needed, most enzymes are not commercially produced. Therefore, despite the high potential for application as reduced calorie sweeteners, limited research exists on the immobilization of these enzymes. d-tagatose 3-epimerase from *Pseudomonas* sp. ST-24 was immobilized on porous chitosan beads and produced d-sorbose from d-tagatose in buffer. The immobilized enzyme retained activity over 5 cycles, with ~70% conversion achieved after each cycle.^53^ Similarly, a thermostable l-arabinose isomerase from *Escherichia coli* was immobilized in alginate and converted galactose in buffer to tagatose. The immobilized enzyme yielded 42 g/L more tagatose than the free enzyme, and achieved an overall conversion of 46%.^54^ Recently, d-psicose 3-epimerase from *Agrobacterium tumefaciens* has been immobilized on graphene oxide to produce d-psicose in buffer from d-fructose, but only retained ~20% activity after 10 cycles of reuse.^55^ Valorization of lactose into rare sugars is likely to be an emerging area of research in immobilized enzyme systems, with efforts focusing on improving conversion efficiency and activity retention with repeat use.

In addition to enzymatic conversion of lactose into sweeteners, lactose can be converted through transglycosidation to produce health beneficial prebiotics including lactulose, lactosucrose, and galacto-oligosaccharides. Lactulose (4-*O*-β-d-galactopyranosyl-d-fructose) is a disaccharide formed from the isomerization of lactose and can be produced through the transgalactosylation of lactose using β-galactosidase and fructose as a co-substrate. In one work, β-galactosidase from *Kluyveromyces lactis* was immobilized on magnetic chitosan microspheres and combined with a commercially available immobilized glucose isomerase (from Novozymes*)*. The researchers demonstrated the importance of characterizing the whole system as it was determined that fructose needed to be added to the feed stream to enable lactulose production and transglycosidation was further promoted by reducing feed stream water activity.^56^ Similarly, β-galactosidase (from *K. lactis)* immobilized on activated silica gel was investigated for both batch and continuous production of lactulose. While β-galactosidase retained ~50% activity after immobilization, producing 19.1 g/L lactulose, only 52.9% of this activity remained after 10 cycles of reuse.^57^ In other work, β-galactosidase from *K. lactis* immobilized on silica gel was used in tandem with glucose isomerase from *Streptomyces rubiginosus* immobilized on silica gel to produce lactulose. The immobilized multi-enzyme system retained 57.1% activity after seven cycles, and produced 7.68 g/L lactulose.^58^ These results suggest the need for further research in the search for β-galactosidase compatible immobilization methods to provide an economic advantage to enzymatic processing of lactose-containing dairy waste streams.

Galacto-oligosaccharides are another important class of prebiotics that have been produced from lactose in dairy waste streams. Li et al. prepared cross-linked enzyme aggregates (CLEAs) of β-galactosidase (expressed in *Escherichia coli*) with enhanced stability under pH and thermal stress and retention of ~82% activity after ten cycles of reuse in production of galacto-oligosaccharides. The authors suggested that the novel enzyme, isolated from a marine metagenomic library, had an increased tolerance for galactose, and that immobilization enhanced transglycosidation activity.^59^ Similarly, Banjanac et al.^60^ immobilized β-galactosidase (from *Aspergillus oryzae*) on silica nanoparticles and observed an increase in transglycosidation activity. Galacto-oligosaccharides production increased from 30 g/L/h using free enzyme, to 90 g/L/h using immobilized β-galactosidase, supporting the potential for immobilized enzyme systems in enhancing enzyme performance.^60^ Recently, Eskandarloo and Abbaspourrad immobilized β-galactosidase (from *Aspergillus oryzae*) on functionalized glass beads for the production of galacto-oligosaccharides from whey permeate. After two cycles of flowing whey permeate through the packed-bed reactor, the authors reached a maximum conversion rate of 39.3%, with only a 4.6% reduction in galacto-oligosaccharides yield after eight cycles, suggesting durability of the immobilized catalyst.^61^ Finally, the prebiotic lactosucrose (*O*-β-d-galactopyranosyl-(1–4)-*O*-α-d-glucopyranosyl-(1–2)-β-d-fructofuranoside) has been synthesized using β-galactosidase (from *Bacillus circulans*) immobilized on chitosan microspheres. The authors determined that higher operating temperatures (50 °C, 64 °C) favored the production of galacto-oligosaccharides, while lower temperatures (30 °C) favored lactosucrose production, highlighting the importance of process control. The microspheres showed no losses in lactosucrose yield after 30 cycles. These results also demonstrate the ability to tailor use of immobilized enzyme systems to optimize profit, as a single enzyme can potentially produce multiple products depending on the processing conditions.^62^ The aforementioned examples demonstrate the versatility of β-galactosidase to produce a range of value-added products, and the importance of characterizing the influence of the immobilization method on the stability of the enzyme after many cycles of reuse.

While the greatest body of research has explored the influence of support material, tether molecule, and cross-linking reagents on immobilization of β-galactosidase, opportunities in synthetic biology offer alternative approaches which permit site-directed orientation during immobilization, production of the enzyme in an extremophile, or stabilization of subunit interfaces to prevent their denaturation during immobilization. For example, fusion tags are site-specific binding proteins that can be engineered into an enzyme, permitting cross-linker free, site-directed immobilization. A cellulose binding domain has been genetically engineered into β-galactosidase (from *Thermoanaerobacter ethanolicus* and *Lactobacillus bulgaricus* L3) for immobilization onto microcrystalline cellulose and application in lactose hydrolysis.^63,64^ After 10,000 column volumes were passed through the system, no decrease in hydrolysis efficiency was observed in the β-galactosidase from *Thermoanaerobacter ethanolicus*. This cellulose binding domain allowed for β-galactosidase to bind to microcrystalline cellulose and resulted in 49% yield in the production of galacto-oligosaccharides.^64^ After 20 cycles of reuse, the immobilized β-galactosidase from *Lactobacillus bulgaricus* L3 retained over 85% activity.^63^ These results show promise for biological modifications in design of immobilized enzyme systems to permit use inexpensive carriers with potential for improved activity retention over multiple cycles.

Opportunities exist for the valorization of lactose-rich dairy waste streams using immobilized enzyme systems, but further research is necessary to evaluate these systems in practical systems, such as whey permeate, and to increase stability over multiple cycles to offset the cost of immobilized enzyme systems. Importantly, while research in the valorization of monosaccharides and disaccharides present in food processing waste streams has largely focused on lactose present in whey, whey permeate, and acid whey, the immobilized enzymes could be applied in other applications with similar substrates present, such as sweetener wastewater treatment or fruit processing waste streams.

### Polysaccharides

Polysaccharides account for a significant portion of waste streams from processing of fruit, vegetable, and crustacean products, yet are valuable substrates for enzymatic conversion. For example, potato processing wastewater, containing starch, can have a biological oxygen demand upwards of 5000 mg/L, which is unable to be released into wastewater without treatment.^65^ Enzymes such as amylases, cellulases, and xylanases can transform such polysaccharides-rich waste streams into value-added products including sweeteners, bioplastics, biofuels, and prebiotics. In this section we describe opportunities for valorization of starch, cellulose, and chitosan waste streams. Starch-rich waste streams originate from the processing of corn, rice, potato, and sweet potato products. Indeed, it is estimated that during potato processing, 16% of the starch is lost during washing and slicing.^66^ Yokoi et al.^67^ further reported that dried powder from sweet potato processing contained ~50% starch, representing another opportunity for enzymatic conversion. The most common method to utilize starch is through hydrolysis to monosaccharides, although opportunities exist in lipase-catalyzed acylation to produce texture modifiers and plasticizers, as reviewed by Alissandratos and Halling.^4^ Briefly, starch acylation can be performed with chemical catalysts,^5^ but enzymatic routes offer enhanced specificity and milder reaction conditions.^4^ Immobilized enzyme systems have been designed to improve enzyme stability in solvents necessary to permit solubility of both starch and acyl donor substrates.^4^ Immobilized enzymes have also been used for starch hydrolysis reactions in an attempt to increase yield and processing efficiency. Gupta et al. separately immobilized α-amylase (from *Bacillus licheniformis*) and amyloglucosidase (from *Aspergillus niger*) via adsorption on ion-exchange resin beads, and hydrolyzed starch to produce 96 dextrose equivalent syrup at high concentrations to mimic industrial processing conditions. While temperature stability increased upon immobilization, the time required for conversion increased, suggesting the need for further improvement in the immobilization process.^68^ In other work, Talekar et al.^69^ co-immobilized α-amylase (CLARASE® L- 40,000 from Riddhi Siddhi Gloco Biols Ltd.), glucoamylase (OPTIDEX® L-300 from Riddhi Siddhi Gloco Biols Ltd.), and pullulanase (OPTIMAX® L-300 from Riddhi Siddhi Gloco Biols Ltd.) in a carrier-free method using glutaraldehyde. The researchers compared batch hydrolysis of corn starch by the free enzymes, individually immobilized cross-linked enzyme aggregates (CLEAs), and combined cross-linked enzyme aggregates (combi-CLEAs) which immobilized all three enzymes in the same CLEA. When exposed to the three enzymes in soluble form, 40% of the starch was hydrolyzed, while CLEAs hydrolyzed 60% of the starch, and combi-CLEAs hydrolyzed 100% of the starch under the same conditions. In addition to complete conversion of starch, co-immobilization enhanced the temperature and pH stability of the enzymes, and allowed for 5 cycles of reuse with no losses in activity, suggesting promise for industrial use.^69^ The same group also studied the same cocktail of enzymes (α-amylase from Thermo-Nzyme L and glucoamylase from Gluco-Nzyme L from Enzymes India Pvt. Ltd., and pullulanse from Riddhi Siddhi Gloco Biols Ltd.) immobilized on amino-functionalized iron oxide nanoparticles, and saw similar results as the combi-CLEAs: tri-immobilized enzymes achieved faster and better conversion rates than single-immobilized and free enzymes, with no observed loss in activity after 8 cycles of reuse.^70^ These results suggest that co-immobilized enzymes may help increase reaction yield, and should be considered when designing immobilized enzyme systems for valorization processes which require multiple biotransformations.

Cellulosic materials have also been studied as polysaccharide substrates for enzymatic valorization because of their potential to be hydrolyzed into monosaccharides, which can be used directly as sweeteners or further transformed into products such as biofuels, surfactants, and rare sugars with the help of additional enzymes or microorganisms. Lignocellulose, composed of a complex mixture of lignin, celluloses, and hemicelluloses, is the primary component in sugarcane bagasse and field crop stover, and due to its chemical complexity requires multi-enzymatic systems for valorization. In one example, Periyasamy et al.^71^ co-immobilized cellulase, xylanase and β−1,3-glucanase (from *Trichoderma citrinoviride* AUKAR04) in a combi-CLEA, and increased hydrolyzation of sugarcane bagasse into monosaccharides by 10% using combi-CLEAs over free enzymes. These combi-CLEAs were also reusable and retained 90% activity through 6 cycles of reuse.^71^ Similarly, Bhattacharya and Pletschke^72^ produced combi-CLEAs of xylanase and mannanase (from *Bacillus gelatini* ABBP-1, *Bacillus licheniformis* ABBP-2, *Bacillus licheniformis* ABBP-3, and *Paeni-bacillus dendritiformis* ABBP-4) to hydrolyze lignocellulosic waste from sugarcane bagasse and milled corn stover. Both applications showed a greater than 150% higher conversion than free enzymes.^72^ β-glucosidase has been explored as a way to drive cellulose hydrolysis to completion through hydrolysis of the intermediate, cellobiose, into two glucose units.^73^ β-glucosidase has been immobilized on a variety of materials including nanoparticles,^74^ acrylic polymers,^75,76^ and biopolymers.^77^ In one report, β-glucosidase (from *Aspergillus niger*) was immobilized in calcium alginate and no losses in activity were observed over 20 cycles, while in another report, β-glucosidase (from *Aspergillus niger*) was immobilized with glutaraldehyde on iron oxide nanoparticles with ~50% reduction in activity after 16 cycles.^74,77^ In other work, Borges et al.^76^ immobilized β-glucosidase (from *Trichoderma reesei*) using two different methods: multipoint covalent attachment on glyoxyl-agarose and carboxyl activated adsorption on polyacrylic resin. The adsorbed β-glucosidase was applied to sugarcane bagasse, and outperformed the covalently attached β-glucosidase, suggesting impairment of the enzyme from the covalent linkages.^76^ These results highlight the importance of both carrier choice and immobilization method in designing an immobilized enzyme system. Further improvements to lignocellulosic material upcycling can be addressed through proper selection of the immobilization process and modification of cellulose, lignin, and cellobiose degrading enzymes.

Pectic substances (pectic acid (polygalacturonic acid) and pectin (methyl-esterified polygalacturonic acids)) can be extracted from fruit and vegetable waste streams and are well utilized as thickeners, texturizers, fillers, and glazes. Using techniques similar to that described for lignocellulose valorization, as well as those reported for juice clarification, immobilized enzymes can add value to alternative underutilized pectin-rich waste streams (e.g., onion skins).^78^ In one work, pectinase from *Bacillus licheniformis* was entrapped in agar-agar with activity retention for up to 10 cycles of reuse.^79^ In other work, pectin-derived monosaccharides galacturonic acid and arabinose were produced from sugar beet pulp using a mixture of pectinases (Enzyme V from Novozymes and enzyme E from Genencor) at a release rate of 79% and 82%, respectively.^80^ A recent report utilized a 2^4^ full factorial central composite design to optimize cross-linker concentrations (polyethylenimine and glutaraldehyde), loading time, and enzyme concentration in immobilization of pectinase on alginate-agar gel beads. Thermostability and activity retention upon reuse was improved by immobilization, and correlations between immobilization parameters and corresponding yields highlighted the value of using full factorial design in enzyme immobilization research.^81^ Immobilized enzymes therefore present an opportunity in deconstructing pectin into pectin-derived monosaccharides (galacturonic acid and arabinose) for use as biofuel feedstocks or as building blocks into bioplastics as well as pectin-derived prebiotic oligosaccharides.

Another polysaccharide waste stream of potential value is chitin, from fungi and crustaceans, which can be deacetylated to yield the biopolymer chitosan. Chitosan can further be hydrolyzed into chitooligosaccharides, which have improved solubility and enhanced biological activities (e.g., antimicrobial, antioxidant, prebiotic properties).^82^ In one study, α-amylase (from *Bacillus amylolyquefaciens*) was immobilized on glyoxyl-agarose beads, retaining ~25% activity compared to free enzyme. The immobilized enzyme was able to convert 73% of the chitosan to chitooligosaccharides following exposure to the catalyst first in a continuous stirred tank reactor before flowing through a packed-bed reactor. However, relative activity of the immobilized α-amylase decreased by ~40% after two days exposure to reaction conditions optimal for chitooligosaccharide production (i.e., 50 °C in 50 mM acetate buffer pH 5.0).^83^ The pathways outlined for valorization of cellulosic, lignocellulosic, pectin and starch wastes (in agricultural sources noted here as well as others containing the same polysaccharides) therefore have the potential for biotransformation of value-added products by immobilized enzymes.

### Lipids

**Fig. 4:**
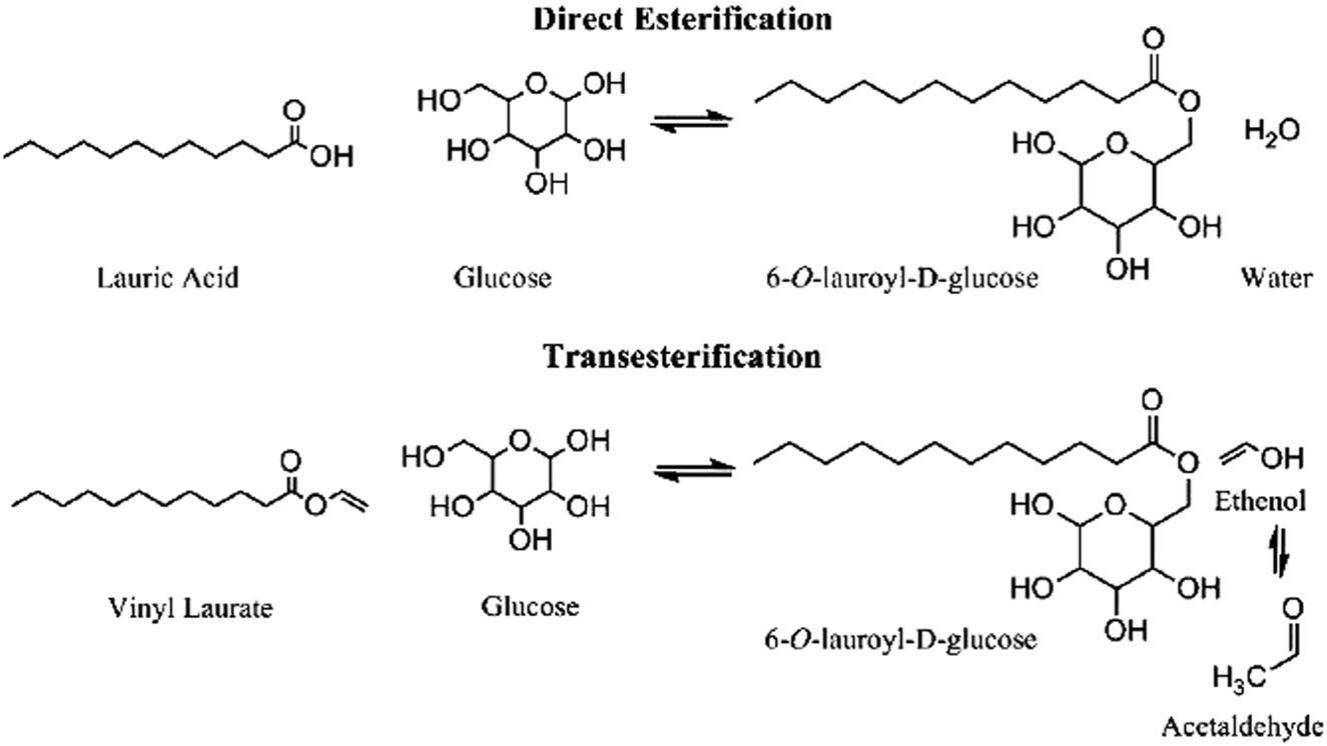
Example of esterification reactions performed by lipase to produce value-added products from fatty acids. Reprinted with permission from Andler et al.^37^ Copyright 2017 American Chemical Society

The potential environmental and economic impact of waste lipid valorization is significant: over 2 billion pounds of yellow grease (i.e., waste cooking oil) were produced in the United States in 2015.^84^ Waste oil can be enzymatically transformed into value-added products such as biodiesel, surfactants (Fig. 4), and lubricants. Of these applications, the enzymatic production of biodiesel from waste oil using lipases is the most widely studied. As with most enzymes, lipases can be produced from many microbial sources with varying impact on performance. For example, lipases from *Thermomyces lanuginosus* and *Candida antarctica* B were sequentially used for hydrolysis and esterification, respectively, to produce biodiesel from waste cooking oil. Over 90% conversion was achieved after 10 h hydrolysis and 10 h esterification; however, after 5 cycles of reuse, lipase from *Thermomyces lanuginosus* lost activity after each cycle, while lipase from *Candida antarctica* B retained activity throughout the study.^85^ In other work, *Rhizomucor miehei* lipase and *Candida antarctica* lipase immobilized on epoxy-functionalized silica particles were applied to increase the yield of biodiesel from waste cooking oil, with 91.5% conversion achieved after 10 h.^86^ Commercial immobilized lipases are available and have also been evaluated for biodiesel production from waste oil, most commonly lipases immobilized on acrylic resin (Novozym 435)^87–90^ and silica gel carriers (Lipozym TL IM).^91,92^ These commercially available lipases have been applied to biodiesel production in solvent-free systems,^87,89,90^ packed-bed reactors,^89^ and with ultrasound assistance.^90–92^ While these immobilized enzymes show enhanced activity and allow for reuse, studies have shown that the support material can further improve the activity of commercially available enzymes through enzyme–material interactions.^93,94^ Recent research on immobilized lipases for waste oil valorization commonly explores silica supports,^86,94–96^ resins,^97–100^ biological supports (e.g., chitosan),^101,102^ and nanomaterials.^103–107^

Recent research on biodiesel production from lipases immobilized on mesoporous silica has emphasized the influence of support material on enzyme performance. As mentioned previously, optimization of the material is important to ensure active sites are not blocked, yet prevent the enzyme from migrating from the support. Zhang et al. compared mesoporous silica supports of varying channel sizes (1.8, 14.0, and 28.0 nm) for lipase (Lipase LVK-S200 from LEVEKING Co. Ltd.) immobilization, and observed an optimal 80.1% yield of biodiesel from unrefined waste cooking oil from the mid-range 14 nm channel size. The authors concluded that while increasing channel size improves specific activity to a point, this only holds true up to an optimal channel dimension. Interestingly, it was also observed that the need for channel size optimization was conditional upon the nature of the feedstock: the more complex nature of waste cooking oil (with insoluble materials which may block smaller channels) benefitted from channel size optimization while pure olive oil was less sensitive to channel size.^95^ In other work, Ferrero et al.^96^ achieved 89% and 91% biodiesel yield from soybean oil and sunflower oil, respectively, using a modified mesoporous silica as a support material for lipase (from *Pseudomonas fluorescens*). Waste cooking oil was also studied as a substrate, and yielded similar results as the neat oils (90% yield), proving the specificity of lipase in the presence of complex matrices. However, recovery and reuse experiments are necessary to determine long term stability and reuse.^96^

Lipases have also been immobilized on polymeric resins for production of biodiesel, fatty acids, and surfactants from waste oils. Lopresto et al. immobilized lipase (from *Pseudomonas cepacian*) on an epoxy-acrylic resin with 46% biodiesel yield after initial use. However, the yield dropped to ~22% after the 6th cycle of reuse, suggesting the need for further optimization of the support material in the stabilization of lipase.^98^ Other research has studied lipase (Lecitase Ultra from Novozymes) immobilized on various styrene divinylbenzene resins to produce monoacylglycerols from acidic palm oil free fatty acid residues. Immobilized lipase yielded 50–60% conversion under continuous-flow conditions; however, some resins lost ~30% protein after the 4th cycle of reuse. Increased protein migration and subsequent loss of activity after repeated used correlated with pore size of the styrene divinylbenzene copolymer, in which enzymes more readily leached through larger pores.^99^ Lipase (from marine *Streptomyces* sp. strain W007) was also immobilized on a commercial styrene divinylbenzene resin (XAD1180), with a 95.45% yield of biodiesel when exposed to waste cooking oil for 24 h. After 4 cycles, 70% activity remained, showing promise for reusability. Importantly, this immobilized lipase system retained activity in the presence of high relative concentrations of methanol, a necessary component in the feedstock of lipase-mediated biodiesel production. The reported methanol-tolerant immobilized lipase had an impressive biodiesel yield of 89.5%, while the three commercially available lipases (Novozym 435, Lipozyme TL IM, and Lipozyme RM IM) presented less than 10% yield (9.32%, 5.8%, and 2.79% yield, respectively).^100^ Another study stabilized lipase (from *Malassezia globosa*) on an epoxy-functionalized resin to convert free fatty acids to fatty acid methyl esters in a high-acid content waste cooking oil, while leaving the triglycerides in solution. After 8 h, lipase was able to reduce the free fatty acid content from 28.69 to 0.05%. After the biotransformation, the remaining triglycerides were chemically converted to fatty acid methyl esters, resulting in a final conversion of 98.24%.^97^ These results further demonstrate the specificity of lipases to perform in complex matrices, and show promise for combined technologies to further increase reaction yields.

Lipases show promise for the conversion of waste cooking oil to biodiesel, but further optimization of support materials and experimental design is necessary to achieve high conversion rates and activity retention over multiple cycles. While immobilized enzyme cost can be a barrier to commercial adoption, a recent cost analysis of enzymatic biodiesel production suggested that the biggest cost hurdle is the cost of the support material, and not the enzyme itself.^108^ However, as mentioned earlier, increased activity over repeated cycles can provide catalysts that are less expensive than commercially available lipases in the market.^86^ Therefore, further research is necessary on renewable resources for enzyme immobilization.

### Proteins

Protein waste from the food industry can come from a variety of sources including, but not limited to, dairy, oilseeds, grains, soybeans, and eggs. Proteins from waste streams can be hydrolyzed by proteases into bioactive peptides or commodity chemicals (e.g., polymer precursors).^109^ Enzymatic routes are favorable over chemical processes, which lack facile control, as seen by the destruction of tryptophan during acid hydrolysis.^109,110^ Dairy waste, specifically whey protein, has been hydrolyzed by immobilized trypsin. Mao et al. immobilized trypsin (Type I from bovine pancreas) on porous, polymethacrylate monoliths with a pore size of 2.1 µm, and achieved a degree of hydrolysis of 9.68%. The degree of hydrolysis of free trypsin reached ~6% under the same conditions, suggesting favorable interactions upon immobilization. Importantly, the peptide analysis differed between the immobilized trypsin and the free trypsin, demonstrating the impact of immobilization methods on enzyme selectivity.^111^ Trypsin has also been immobilized on renewable supports for whey protein hydrolysis, including spent grain and lignocellulose.^112,113^ Multipoint covalent attachment was used to tether trypsin (from porcine pancreas) to spent grain, and retained greater than 90% activity after 5 cycles of reuse, as measured by the synthetic substrate, *N*-α-benzoyl-dl-arginine-*p*-nitroanilide. In contrast, trypsin adsorbed to the spent grain lost greater than 50% activity after 5 cycles of reuse, likely due to migration of the enzyme from the support. Covalently attached trypsin achieved similar degrees of hydrolysis compared with free trypsin (6.5% and 6.3%, respectively), but analysis of the smaller peptides and larger proteins showed that immobilized trypsin preferred larger substrates. Thus, analysis of the products, in addition to the overall product conversion is important to consider when working with trypsin, as immobilization can alter enzyme selectivity.^112^ Trypsin (source not disclosed) has also been immobilized on a renewable support for whey protein hydrolysis using lignocellulose, specifically, corn cob powder. Trypsin was immobilized using two methods: glyoxyl activation and iminodiacetic aldehyde-glyoxyl activation and produced a degree of hydrolysis of 15.46% and 12.49%, respectively. Additionally, the trypsin-glyoxyl system was applied in a packed-bed reactor, and obtained, on average, 23% degree of hydrolysis. These results further demonstrate how immobilization methods, as well as process parameters, can influence enzyme activity. Whey protein was also hydrolyzed to form antioxidant peptides using glutaraldehyde-agarose immobilized aspartic protease from *Salpichroa origanifolia* fruit. The immobilized protease reached a similar degree of hydrolysis as free enzyme, but was more selective towards α-lactalbumin than β-lactoglobulin. The authors suggested that immobilization can alter the cleavage affinity of the enzyme.^114^ These results further support that immobilization can alter the selectivity of the enzyme, thus potentially impacting the final product.

Soy protein has also been studied as a substrate for waste valorization by proteases. In one study, alcalase alkaline protease (from *Bacillus licheniformis*) was immobilized on magnetic nanoparticles, coated in chitosan and was used to hydrolyze soy protein isolate. The immobilized protease displayed a degree of hydrolysis of 18.38% compared to 17.5% by free enzyme.^115^ However, another study immobilized alkaline protease (from *Bacillus subtilis*) on magnetic nanoparticles and hydrolyzed rapeseed meals, and the degree of hydrolysis of the immobilized enzyme was lower than the free enzyme (9.86% and 10.41%, respectively), suggesting that similar nanomaterial choice is not the sole predictor of stability. Rather, methods of immobilization may cause enzymes to behave differently depending on the source of the enzyme and the substrate.^116^ Alkaline protease (from *B. licheniformis*) was also immobilized on magnetic nanoparticles for the production of oat polypeptides. Under smaller scale conditions (10 mL batch size) 8.4 mg/mL oat polypeptides were produced, but under larger scale conditions (250 mL batch size) only 8.1 mg/mL oat polypeptides were produced. These results, while not practically significant, highlight the importance of scale-up of immobilized enzyme systems, as processing parameters may influence enzyme activity.^117^ Higher degrees of hydrolysis has been achieved through the use of multi-enzyme systems. Pedroche et al.^118^ immobilized trypsin (bovine, from Sigma Aldrich), chymotrypsin (from Sigma Aldrich) and carboxypeptidase (from Serva) on glyoxyl-agarose supports, and was able to achieve a 36% degree of hydrolysis when sequentially applied to *Brassica carinata* protein isolate.

Enzymatic debittering and allergen degradation of protein hydrolysates represents another important opportunity for improving the value of food processing by-products. Indeed, recent work has explored using enzyme assisted degradation of bitter compounds^119–122^ and allergens^123–125^ in protein-rich waste streams (e.g., porcine plasma, soy protein isolate) to increase their value as nutritional supplements and flavor modifiers. While there are some reports on immobilization of alcalase for allergenicity reduction in whey and egg white protein^126,127^ and others on immobilization of animal-derived peptidases for casein and soy protein debittering,^128,129^ improving the catalytic and process efficiency of enzyme-mediated debittering and allergen degradation processes via enzyme immobilization technologies remains an under-researched area for enzyme-mediated valorization of protein-rich waste streams. While examples of immobilized proteases and peptidases applied to food waste streams have been described, opportunities exist to further improve upon the degree of hydrolysis through optimization of immobilization methods.

### Challenges and perspectives

Valorization of food processing waste streams using immobilized enzyme systems presents a unique technological approach to increase the environmental and economic sustainability of food production. Yet, challenges remain which demand additional research to promote their commercial translatability. A major challenge is the numerous available methods of immobilization, coupled with varied operational requirements in terms of waste stream conditions, and the practical challenge that different enzymes behave differently upon immobilization, making it impractical to identify a short list of ideal methods of immobilization for all systems. Another significant hurdle to the implementation of immobilized enzyme systems in waste valorization is cost. An estimated 47% of the cost of an immobilized enzyme system is associated with the support material. Furthermore, the use of purified enzymes instead of whole-cell or crude extract increases the cost of biocatalysis significantly.^130^ Therefore, inexpensive carriers or carrier-free immobilized enzyme systems such as CLEAs must continue to be explored, along with immobilized enzyme systems that utilize whole-cell or crude extracts. As with many technologies that seek to improve environmental sustainability, from waste valorization to anaerobic digesters to solar panels, the cost:benefit ratio shifts with many factors. For food waste stream valorization, the major drivers will be cost of the immobilized enzyme system itself as well as pricing and demand for naturally derived ingredients. An in-depth survey of the economics of waste stream valorization with new immobilized enzyme technologies would be of great value to researchers and industry alike.

Emerging tools in synthetic biology, in which enzymes are genetically modified to incorporate tags that enable site-directed immobilization, improve stability in non-ideal environments, or stabilize subunit interfaces against denaturation, offer new approaches to improving immobilized enzyme performance. Incorporation of a cellulose binding domain in lactase permitted its immobilization onto microcrystalline cellulose.^64^ As cellulose and cellulose derivatives are relatively inexpensive carriers, this approach has the added benefit of reducing the cost of the immobilized enzyme system, further increasing the potential for commercial feasibility. In addition to utilizing low-cost carrier materials, immobilized enzyme systems must retain activity over multiple cycles to decrease the cost per use. While many studies report retained activity with potential for reuse, many immobilized systems show a significant decrease in activity after multiple cycles. While no doubt tedious, rigorous experimental characterization of the stability of immobilized enzyme systems against both activity loss and leaching is necessary to demonstrate the true behavior of these systems in an industrial application.

Because of the complexity of applications of immobilized enzyme systems in food waste valorization, interdisciplinary teams are necessary to drive innovation. For example, the source of the enzyme must be considered when applying enzymes to food applications. The organism used to produce the enzyme must be regulated as GRAS or approved as a processing aid to be used in production of a food ingredient, in accordance with 21 CFR Part 173. Further, while genetically modified enzymes can offer improved performance, the resulting products cannot be deemed certified organic. Indeed, collaborations between experts in biochemistry, molecular biology, genetic engineering, food science, process engineering, agricultural economics, and food law/regulatory are critical in applications driven enzymology research for waste stream valorization.

To improve their likelihood of commercial adoption, immobilized enzyme systems should be designed for implementation into existing processes. For example, many waste streams must be remediated of substances (e.g., phenolic inhibitors, bitter components) prior to serving as compatible feedstocks for enzymatic valorization processes. Multi-enzyme systems also show promise for enhancing conversion rates, which can allow for single-enzyme processes to more effectively catalyze value-added processes. Throughout the research process, the nature of the system must be considered; biological replicates conducted in commercially translatable conditions are necessary to more accurately predict the potential for commercial adoption, as biological systems inherently have variability. In addition, thorough characterization of immobilized enzyme kinetics and diffusion-reaction mechanisms would improve the fundamental understanding of the practical applications of immobilized enzyme systems. Further, commercially applicable conditions are more complex than neat synthetic substrate in buffer and can influence enzyme stability and performance under true conditions. This phenomenon is particularly true for multi-enzyme systems, in which sensitivity to inhibitors, water activity, solvents, and their needs for co-factors or co-substrates must be considered for each enzyme. Because of the complexity of the system (i.e., variability from enzyme source, support material, processing conditions) it is difficult to compare technologies from a specific enzyme/process to another. For example, while lipases are stabilized at a hydrophobic interface, other enzymes will lose activity. Therefore, rational design (e.g., choosing a stabilization method based upon the understanding of the enzyme source, commercial application conditions, and immobilization methods) and understanding of biocatalytic systems must be used to successfully employ immobilized enzymes. Importantly, while it is common practice to report enzymes by their proprietary names and/or the company from which they were purchased/donated, authors must provide detailed source information (e.g., full name of the microorganism that synthesized it) in order to permit such rational design of immobilized enzyme systems by the scientific community. These rationally designed biocatalysts have the potential to increase the sustainability of the food industry through the creation of value-added products and have a positive impact on reducing food waste generated from food processing.
